# CIDP: Analysis of Immunomarkers During COVID-19 mRNA-Vaccination and IVIg-Immunomodulation: An Exploratory Study

**DOI:** 10.1007/s11481-023-10058-x

**Published:** 2023-03-16

**Authors:** Martin K. R. Svačina, Anika Meißner, Finja Schweitzer, Alina Sprenger-Svačina, Ines Klein, Hauke Wüstenberg, Felix Kohle, Helene L. Walter, Michael Schroeter, Helmar C. Lehmann

**Affiliations:** 1 Department of Neurology, Faculty of Medicine and University Hospital of Cologne, University of Cologne, Kerpener Straße 62, Cologne, 50937 Germany; 2 Department of Neurology, Städtisches Klinikum Leverkusen, Leverkusen, Germany

**Keywords:** Vaccine Interaction, Immune Neuropathy, Immune Cells, FcγRIIb/CD32b, Cytokines

## Abstract

Availability of COVID-19 mRNA vaccine for patients with chronic inflammatory demyelinating polyneuropathy (CIDP) treated with intravenous immunoglobulin (IVIg) raises the question of whether COVID-19 mRNA vaccine influences disease activity or IVIg-mediated immunomodulation in CIDP. In this exploratory study, blood samples of CIDP patients on IVIg treatment were longitudinally analyzed before and after vaccination with a COVID-19 mRNA vaccine. A total of 44 samples of eleven patients were characterized at four timepoints by ELISA and flow cytometry in terms of immunomarkers for disease activity and IVIg-immunomodulation. Apart from a significantly lower expression of CD32b on naïve B cells after vaccination, no significant alteration of immunomarkers for CIDP or IVIg-mediated immunomodulation was observed. Our exploratory study suggests that COVID-19 mRNA vaccine does not have a relevant impact on immune activity in CIDP. In addition, immunomodulatory effects of IVIg in CIDP are not altered by COVID-19 mRNA vaccine. This study was registered in the German clinical trial register (DRKS00025759).

**Graphical Abstract:**

Overview over the study design. Blood samples of CIDP patients on recurrent IVIg treatment and vaccination with a COVID-19 mRNA vaccine were obtained at four timepoints for cytokine ELISA and flow cytometry, to assess key cytokines and cellular immunomarkers for disease activity and IVIg-immunomodulation in CIDP.

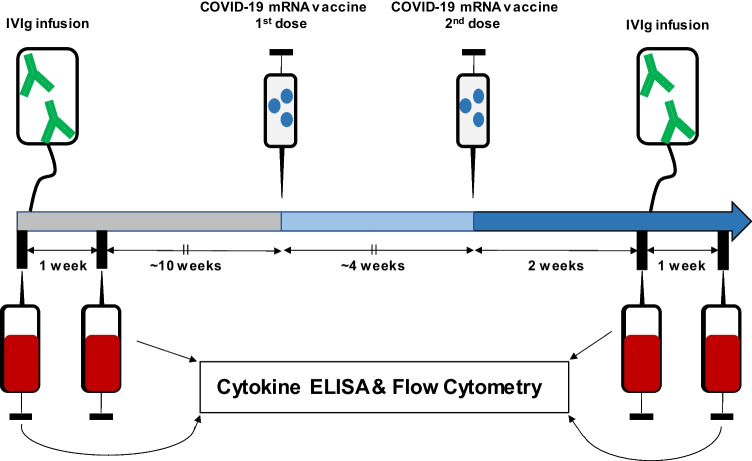

**Supplementary Information:**

The online version contains supplementary material available at 10.1007/s11481-023-10058-x.

## Introduction

Chronic inflammatory demyelinating polyneuropathy (CIDP) is a prototypic autoimmune disease of the peripheral nervous system (Lehmann et al. 2019). Pathomechanisms of CIDP include autoreactive T cells (Chi et al. 2010), impaired B cell maturation and reduced expression of regulatory Fc gamma receptor IIb (FcγRIIb, CD32b) on B cells and monocytes, promoting autoantibody production against peripheral nerve myelin and phagocytosis (Press et al. 2003; Tackenberg et al. 2009). Over the last years, several studies tried to establish markers of peripheral blood mononuclear cells (PBMC) and serum cytokines that might reflect disease activity in CIDP. Of those, reduced numbers of CD32b expressing memory B cells and monocytes, as well as increased numbers of CD16^+^ myeloid dendritic cells, were associated with disease activity and/or poor response to immunoglobulin therapy (Tackenberg et al. 2009; Dyer et al. 2016). Other studies demonstrated that CIDP is associated with a reduction of regulatory T cells, as well as with an increase of monocytes (Matà et al. 2007; Sanvito et al. 2009).

Intravenous immunoglobulin (IVIg) is a first-line treatment for CIDP (Van den Bergh et al. 2021). IVIg attenuates CIDP-mediated autoimmunity via anti-idiotypic antibodies (Svačina et al. 2019), reduction of pro-inflammatory cytokines and chemokines (i.e., interleukin-6 [IL-6], or macrophage inflammatory protein-1α [MIP-1α], Hartung 2008; Ritter et al. 2014; Kajii et al. 2014), and by enhancing the expression of CD32b receptors on B cells and monocytes (Tackenberg et al. 2009). The effects of IVIg on CD32b expression are time-dependent. Whereas one week after IVIg infusion, CD32b expression decreases on B cells and monocytes due to IVIg-induced receptor internalization (Bouhlal et al. 2014; Dyer et al. 2016), Tackenberg and colleagues demonstrated that CD32b expression on these cells increases two to three weeks afterward (Tackenberg et al. 2009). Furthermore, IVIg suppresses pro-inflammatory myeloid dendritic cells and their pro-inflammatory CD32a (FcγRIIa) receptors (Boruchov et al. 2005; Dyer et al. 2016).

COVID-19 mRNA vaccine is known to provoke an immune response characterized by a transient increase of pro-inflammatory cytokines (e.g., IL-6), resulting in a SARS-CoV-2 specific proliferation of T cells, B cells, and anti-SARS-CoV-2 antibody-producing plasma cells (Goel et al. 2021; Jordan 2021). In contrast, whole cell counts of naïve, non-naïve, or memory B cells are considered to remain stable across the time course of vaccination (Goel et al. 2021).

Possible adverse effects of COVID-19 mRNA vaccine, like aseptic (peri-)myocarditis, might be caused by a misdirected, possibly stimulus-independent immune response after vaccination, but their exact pathomechanism is currently unknown (Vidula et al. 2021).

It is thus conceivable, yet unknown that COVID-19 mRNA vaccine alters the aberrant immune response in CIDP or impacts the immunomodulatory effects of IVIg. Therefore we here longitudinally examined for the first time the expression of selected cellular and humoral immunomarkers of CIDP and of IVIg-immunomodulation during the vaccination with a COVD-19 mRNA vaccine.

## Methods

### Patient Characteristics and Study Design

Eleven patients (9 male / 2 female, mean age 62 ± 17 years) with CIDP participated in this study. Inclusion criteria were confirmed or probable CIDP (according to the EFNS/PNS criteria (Van Den Bergh et al. 2010)) on recurrent IVIg treatment (every four weeks), the absence of autoantibodies against paranodal junction proteins, vaccination with a COVID-19 mRNA vaccine (Pfizer-BioNTech BNT162b2), and written informed consent for study participation. Exclusion criteria were acute infections or intake of immunosuppressants or systemic corticosteroids within the previous three months. Mean baseline INCAT disability score was 2 ± 2, mean baseline Rasch-built overall disability scale (R-ODS) score was 73 ± 19.

Blood serum samples and peripheral blood mononuclear cell (PBMC) samples were collected at four timepoints: Before vaccination (mean 11 ± 4 weeks before the first vaccine dose), samples were collected before (1^st^, on the same day) and one week after IVIg infusion (2^nd^). The mean interval between the first and the second dose of COVID-19 vaccine was 4 ± 1 weeks. Sample collection was repeated two weeks after the second COVID-19 vaccine dose immediately before (3^rd^) and one week after IVIg infusion (4^th^).

### Sample Preparation

Blood samples were collected by venipuncture using Sarstedt S-monovettes® (Sarstedt, Nümbrecht, Germany) for serum collection and the BD Vacutainer CPT™ system (BD Biosciences, San Diego, CA, USA) for PBMC isolation.

Serum samples were obtained by centrifugation of whole blood monovettes at 3000 rotations per minute (rpm) for 10 min and then stored at -20 °C for further use.

PBMCs were isolated from the BD Vacutainer CPT™ system according to the manufacturers ‘ instructions before being stored at -80 °C (15 million PBMCs in 100 µl dimethylsulfoxide [DMSO] and 900 µl fetal bovine serum [FBS]).

### Immunophenotyping by Flow Cytometry

The following antibodies were used for the analysis of peripheral B and T lymphocytes, monocytes, and myeloid dendritic cells by flow cytometry: CD3 (OKT3)-BV650, CD11c (3.9)-BV510, CD14 (63D3)-PE, CD16 (3G8)-APC/Cy7, CD19 (HIB19)-PE/Cy7, CD27 (O323)-BV785, CD32b/c (S18005H)-APC, CD56 (5.1H11)-PE Dazzle 594, all from Biolegend, San Diego, CA, USA, and CD32a (#2)-FITC (SinoBiological, Beijing, China).

Raw flow cytometry data were acquired on a BD LSR Fortessa™ cytometer (BD Biosciences) and data were analyzed using FlowJo v.10.8.0 software (BD Biosciences).

All flow cytometry experiments included isotype controls and fluorescence-minus-one controls. Doublet cells were excluded from analyses based on forward scatter-A and forward scatter-H. For each experiment, the lymphocyte and the monocyte population were gated using forward and side scatter.

Monocytes were characterized by excluding CD3 and CD19 positive cells and the differential expression of CD14 and CD16, and defined as classical (CD14^+^CD16^−^) and non-classical (CD14^dim^CD16^+^) monocytes. Thereafter, the expression of pro-inflammatory CD32a or anti-inflammatory CD32b on monocytes was assessed. The whole lymphocyte population was differentiated into T cells (CD3^+^CD32a^+^ and CD3^+^CD32b^+^), naïve B cells (CD19^+^CD27^−^), either expressing CD32a (CD19^+^CD27^−^CD32a^+^) or CD32b (CD19^+^CD27^−^CD32b^+^), or memory B cells expressing the same antigens (CD19^+^CD27^+^CD32a^+^, CD19^+^CD27^+^CD32b^+^). As CD32c is uniquely expressed on NK cells (Veri et al. 2007), only CD32b expression was considered on the cell populations analyzed in this study.

Myeloid dendritic cells (mDCs) were defined as CD11c^+^ live cells that were negative for CD3, CD14, CD19, and CD56 (Suppl. Figure 1).

### Serum Cytokine ELISA

Serum samples were analyzed for IL-6, IL-10, IL-33, MIP-1α, and TGF-β concentrations using sandwich enzyme-linked immunosorbent assay (ELISA) kits (ImmunoTools, Friesoythe, Germany, for IL-6 and IL-10) and Invitrogen human IL-33, MIP-1α, and TGF-β sandwich ELISA kits (Thermo Fisher Scientific, Waltham, MA, USA) according to the manufacturers ‘ instructions. Plates were read at 450 nm and optical densities were analyzed using the BMG Labtech MARS® software (BMG Labtech, Ortenberg, Germany). Serum cytokine concentrations were calculated by interpolating the individual sample’s optical density with a standard concentration curve.

### Statistical Analysis

Statistical analysis was performed using GraphPad PRISM 9.0 software (Graphpad, San Diego, CA, USA). Normality was tested using D’Agostino and Pearson omnibus normality test, before either multiple paired t-tests or Wilcoxon tests were performed. A p-value < 0.05 was considered statistically significant.

## Results

All CIDP patients showed relevant IgG reactivity against SARS-CoV-2 two weeks after the second dose of COVID-19 mRNA vaccine, which was measured by chemiluminescent microparticle immunoassay (CMIA, mean anti-SARS-CoV-2 IgG serum titer: 1670 ± 914 BAU/ml).

**Fig. 1:**
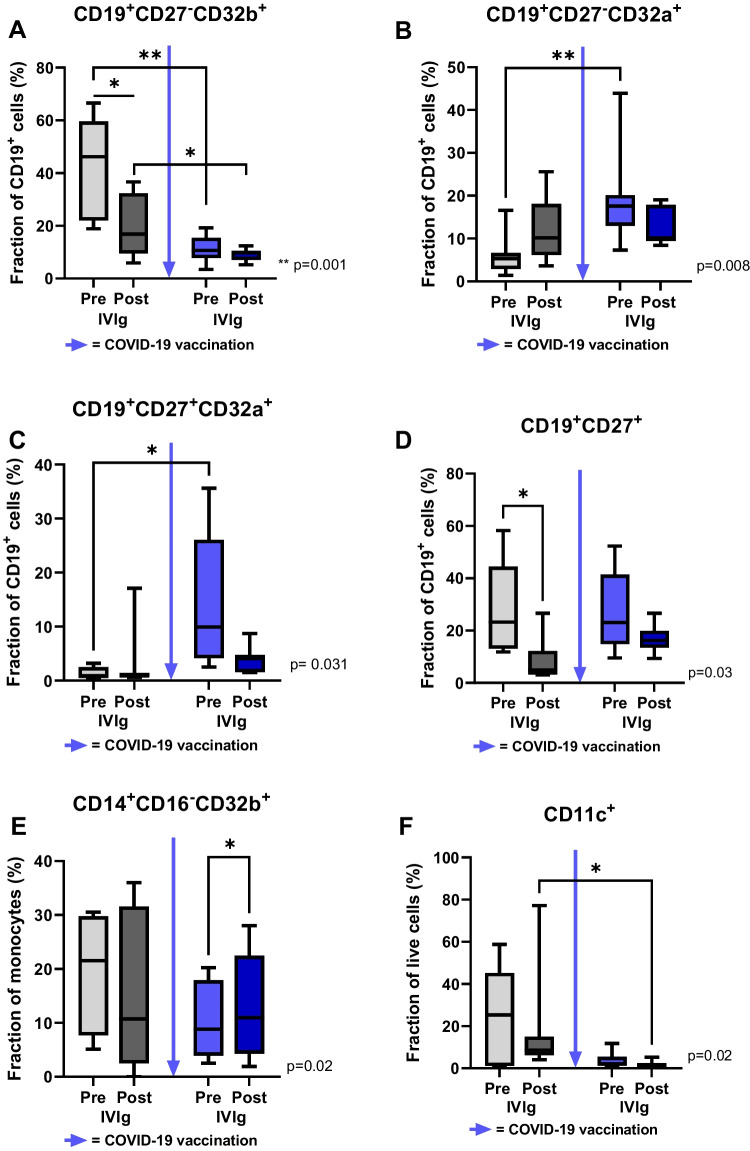
Alterations of immune cell fractions after IVIg administration before and after COVID-19 vaccination. CD32b expression on naïve B cells is significantly reduced after IVIg administration and after COVID-19 vaccination (**A**). Contrariwise, CD32a expression on naïve and memory B cells increases after COVID-19 vaccination (**B**,**C**). IVIg administration significantly decreases CD19^+^CD27^+^ memory B cells in an unvaccinated state, but not after COVID-19 vaccination (**D**). CD32b expression on classical monocytes is increased after IVIg administration and vaccination (**E**). IVIg administration tends to reduce CD11c^+^ myeloid dendritic cells, with a significantly reduced cell amount after COVID-19 vaccination (**F**). Blue arrow = Second dose of COVID-19 mRNA vaccine. * *p* < 0.05, **p < 0.01

**Fig. 2:**
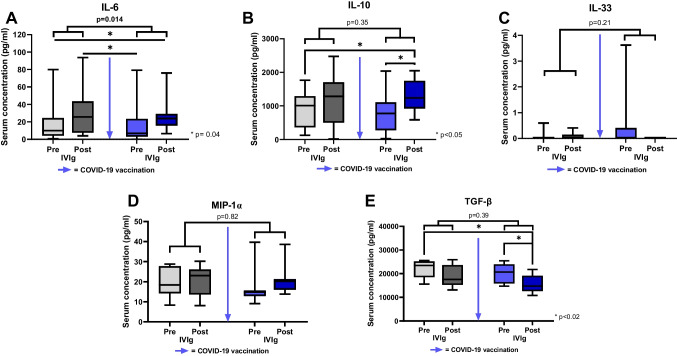
Alterations of serum cytokine concentrations after IVIg administration and COVID-19 vaccination. The relative increase of IL-6 concentrations after IVIg administration is significantly higher after COVID-19 vaccination than in an unvaccinated state, whereas the absolute concentrations remain stable (**A**). IL-10 concentrations significantly increase after IVIg administration and COVID-19 vaccination, but no significant difference in relative increase is seen after vaccination compared to an unvaccinated state (**B**) IL-33 and MIP-1α concentrations are not significantly altered by IVIg administration or COVID-19 vaccination (**C,D**). TGF-β concentrations decrease after IVIg administration, without a relevant influence of COVID-19 vaccination on the extent of the relative decrease (**E**). Blue arrow = Second dose of COVID-19 mRNA vaccine. *p < 0.05

1. **Immunomarkers of CIDP disease activity before and after COVID-19 vaccination**
  CD32b expression on naïve B cells was lower in post vaccination samples (11.2 ± 4.9% vs. 43.8 ± 17.8%, p = 0.001; Fig. 1A), whereas no significant difference was seen on memory B cells (28.2 ± 17.8% vs. 10.6 ± 6.9%, p = 0.06). Contrariwise, in post vaccination samples, CD32a expression on naïve (18.9 ± 10.3% vs. 5.8 ± 4.2%, p = 0.008) and memory (14.7 ± 12.4% vs. 1.3 ± 1.1%, p = 0.03) B cells was higher compared to samples before vaccination (Fig. 1B-C).
  The whole memory B cell fraction (CD19^+^CD27^+^) was not different in samples before and after vaccination (28.0 ± 15.4% vs. 28.4 ± 17.4%, p = 0.81; Fig. 1D). Furthermore, COVID-19 vaccination did not alter CD32b expression on classical monocytes (10.2 ± 6.8% vs. 19.6 ± 11.7%, p = 0.25; Fig. 1E).
2. **Immunomarkers of IVIg-immunomodulation before and after COVID-19 vaccination**
  IVIg administration significantly reduced CD32b expression on naïve B cells before (19.4 ± 11.5% vs. 43.8 ± 17.8%, p= 0.02; Fig. 1A) and after vaccination (9.2 ± 2.3% vs. 11.2 ± 4.9%, p = 0.03; Fig. 1A), whereas CD32b expression on memory B cells was not significantly changed by IVIg treatment (28.2 ± 17.8% vs. 11.3 ± 23%, p= 0.30). IVIg administration did not alter CD32a expression on naïve and memory B cells before (p = 0.16 [naïve B cells], p = 0.66 [memory B cells]) and after (p = 0.25 [naïve B cells], p = 0.06 [memory B cells]) COVID-19 vaccination (Fig. 1B-C).
  IVIg administration resulted in a lower memory B cell amount in vaccine-naïve samples (8.5 ± 8.1% vs. 28.4 ± 17.4%, p = 0.03), but not after COVID-19 vaccination (Fig. 1D). Furthermore, IVIg administration led to a significant increase of CD32b expression on classical monocytes after COVID-19 vaccination (12.6 ± 9.6% vs. 10.2 ± 6.8%, p = 0.02; Fig. 1E).
  The amount of myeloid dendritic cells (mDC) was significantly lower after COVID-19 vaccination and IVIg administration compared to samples prior to vaccination (1.8 ± 1.6% vs. 17.5 ± 24.4%, p = 0.02; Fig. 1F). Finally, COVID-19 vaccination and IVIg administration similarly led to a sustained decrease of CD32b expressing T cells (0.1 ± 0.1% vs. 16.4 ± 19.4%, p = 0.008) and non-classical monocytes (0% vs. 2.3 ± 3.1%, p = 0.03).
3. **Serum cytokine levels before and after COVID-19 vaccination**
  IL-6 was increased in serum samples after IVIg administration before and after COVID-19 vaccination (Δ IL-6 [post IVIg – pre IVIg] after vs. prior to COVID-19 vaccination [in pg/ml]: 11.0 ± 7.4 vs. 2.3 ± 1.9, p = 0.01), whereas the absolute serum concentrations were comparable (17.6 ± 23.6 pg/ml vs. 17.6 ± 24.2 pg/ml; Fig. 2A).
  Serum concentration differences related to IVIg administration (Δ [post IVIg – pre IVIg]) of IL-10 (381.2 ± 295.5 pg/ml vs. 240.5 ± 173.3 pg/ml, p = 0.35; Fig. 2B), of IL-33 (0.07 ± 0.2 pg/ml vs. 0.52 ± 1.1 pg/ml, p = 0.21; Fig. 2C), of MIP-1α (8.2 ± 8.6 pg/ml vs. 5.9 ± 3.6 pg/ml, p = 0.82; Fig. 2D), and of TGF-β (4147 ± 2567 pg/ml vs. 2826 ± 2217 pg/ml, p = 0.39; Fig. 2E) were not different before and after COVID-19 vaccination. Likewise, absolute serum concentrations of these cytokines were not relevantly altered by COVID-19 vaccination (Fig. 2A-E). 
4. **Disability before and after COVID-19 vaccination**
  No changes in disability, measured by the INCAT disability scale (mean INCAT score before and after vaccination: 2 ± 2) and the R-ODS score (mean R-ODS score before and after vaccination: 73 ± 19), occurred after COVID-19 vaccination. Also, no subjective clinical deterioration was reported from CIDP patients after COVID-19 vaccination.

## Discussion

To our knowledge, this is the first study that explored a set of presumed markers of disease activity and IVIg- immunomodulation in CIDP patients who underwent vaccination with the novel mRNA vaccine BNT162b2. As such, our study provides important insights into the vaccine response in a chronic autoimmune condition. Our data indicate that in CIDP, immunomarkers for disease activity and IVIg- immunomodulation were not relevantly altered by COVID-19 mRNA vaccine.

The most striking difference in the expression of disease activity markers before and after vaccination was the lower expression of anti-inflammatory CD32b on naïve B cells and an increased expression of pro-inflammatory CD32a. CD32b (FcγRIIb) is known to control antibody generation upon immunization (Takai et al. 1996). As such, it appears attractive to speculate that this decline could be a systemic vaccine effect. However, this hypothesis would need confirmation by similar observations in other IVIg-dependent and -independent conditions. The reduction of CD32b on naïve B cells could be related to CD32a upregulation, as these two immunoglobulin receptors mediate opposing functions on B cells, probably leading to a relative CD32b downregulation when CD32a is upregulated (Nimmerjahn and Ravetch 2007). Regarding its function for the pathogenesis of CIDP, we assume that the decrease of CD32b on naïve B cells is not clinically relevant for the following reasons: 1) CD32b is known to decline subsequent to IVIg treatment on naïve B cells in CIDP within the first week (Dyer et al. 2016). 2) CD32b expression on memory B cells—which are also considered to be critical for CIDP and its treatment with IVIg (Tackenberg et al. 2009)—was not altered. 3) None of the patients experienced any clinical deterioration upon the vaccination.

Also, the activation of pro-inflammatory CD32a receptors on B cells may reflect a physiological activation of the humoral adaptive immune system for anti-SARS-CoV-2 antibody production, as studies on pneumococcal vaccine showed that CD32a activation plays a crucial role for vaccine response, promoting vaccine-induced antibody generation (Wiertsema et al. 2006). Regarding its role in terms of mediating IVIg efficacy, it has to be noted that although CD32a is known to be suppressed by IVIg, our data and a previous study indicate that this mechanism of action does not seem to play a major role in the pathogenesis in CIDP (Quast et al. 2015). Our observation that none of our patients progressed or relapsed upon the vaccination further supports this conclusion.

Furthermore, other markers for IVIg-immunomodulation in CIDP, like increasing CD32b expression on classical monocytes (Tackenberg et al. 2009) and a decrease of myeloid dendritic cells (Dyer et al. 2016), were not altered after COVID-19 vaccination, further supporting the assumption that COVID-19 mRNA vaccine does not have a relevant impact on immunomarkers for CIDP.

To our knowledge, this study is also the first that explored potential changes in serum cytokine levels subsequent to the vaccination with a COVID-19 mRNA vaccine. Our findings that most of the assessed cytokines showed comparable expression profiles in paired serum samples before and after vaccination further support the notion that the vaccine overall does not impact markers of systemic inflammation. The slightly stronger increase of IL-6 serum levels post IVIg after COVID-19 vaccination could reflect a vaccine-specific immune response (Jordan 2021). This hypothesis is indirectly supported by a recent study that demonstrated that patients under anti-IL6 treatment showed lower antibody responses to COVID-19 mRNA vaccine (Picchianti-Diamanti et al. 2021). Whether our exploratory findings are also transferable to other autoimmune diseases has to be explored by future studies, and recent reviews focusing on COVID-19 vaccination and its potential to influence the course of Guillain-Barré syndrome or systemic lupus erythematosus demonstrate that such studies would raise potential interest (Mason et al. 2021; Lahoz Fernandez et al. 2022). Furthermore, vaccine selection decision-making models might be useful to minimize adverse vaccine effects in patients with autoimmune diseases, as they present with different host factors and a possibly different susceptibility to adverse immunological side effects than the average population (Abdelwahab et al. 2021; Mason et al. 2021).

A recent study demonstrated that anti-SARS-CoV-2 antibody generation is not directly altered by IVIg in CIDP, and that a sufficient vaccine efficacy is assumable in CIDP patients, although CIDP-specific immunological host factors have to be considered (Svačina et al. 2022).

Our study has several limitations, as the small cohort size of eleven patients only provides first exploratory data about the interaction of COVID-19 mRNA vaccine with immunomarkers of CIDP and IVIg-immunomodulation, that will require confirmation by larger follow-up studies also including untreated CIDP patients or unvaccinated IVIg recipients as control cohorts, as well as longitudinal data about the effects of booster vaccinations, that were lacking in our study. Secondly, as COVID-19 mRNA vaccine prevents SARS-CoV-2 from entering human cells via the angiotensin-converting enzyme 2 receptor (ACE2R; Gattinger et al. 2022), and as data about a differential expression of ACE2R in CIDP and healthy subjects, that could generally influence vaccine efficacy in CIDP, are currently lacking, future studies also will have to elucidate ACE2R expression in CIDP. Thirdly, our study did not evaluate the response of CD8-positive cyctotoxic T cells or plasma cells to COVID-19 mRNA vaccine due to its focus on distinct immunomarkers of CIDP. As cyctotoxic T cells and plasma cells play an important role by executing vaccine-induced SARS-CoV-2 immunity (Ssemaganda et al. 2022), future studies should also focus on vaccine-induced alterations of these cell populations in CIDP. On the other hand, our study evaluated CD32a/b expression on B cells, that either enhance or reduce plasma cell-mediated antibody generation (Karnell et al. 2014), therefore a concordance of post-vaccine plasma cell alterations to the CD32 receptor expression patterns observed in our study appears assumable.

In summary, considering the exploratory approach of our study and all its limitations, our study suggests that neither the examined immune mechanisms in CIDP nor key immunomodulatory pathways of IVIg are significantly altered two weeks after full COVID-19 immunization, which is a timepoint assumed to be safe for post-vaccine IVIg administration by expert opinions, but was so far lacking confirmative data (Doneddu et al. 2021).

## Supplementary Information

Below is the link to the electronic supplementary material.

Supplementary file1 (PPTX 7687 KB)

## Data Availability

The data of this study are not publicly available due to ethical restrictions. They may be made available on reasonable request (e.g., for replicating procedures and results) by the corresponding author after consultation with the co-authors.

## Glossary

*ACE2R*: angiotensin-converting enzyme receptor 2
*CD*: cluster of differentiation
*CIDP*: chronic inflammatory demyelinating polyneuropathy
*COVID-19*: coronavirus disease 19
*DMSO*: dimethylsulfoxide
*ELISA*: enzyme-linked immunosorbent assay
*FACS*: fluorescence activated cell sorting
*FBS*: fetal bovine serum
*FcγR*: Fc gamma receptor
*IgG*: immunoglobulin G
*IVIg*: intravenous immunoglobulin
*mDC*: myeloid dendritic cell
*ml*: milliliter
*PBMC*: peripheral blood mononuclear cells
*PBS*: phosphate buffered saline
*rpm*: rotations per minute
*SARS-CoV-2*: severe acute respiratory syndrome coronavirus 2
*μl*: microliter
