# Supplementary material for: CIDP: Analysis of Immunomarkers During COVID-19 mRNA-Vaccination and IVIg-Immunomodulation: An Exploratory Study
Source: J Neuroimmune Pharmacol. 2023 Mar 16;18(1-2):208–14. doi: 10.1007/s11481-023-10058-x (PMC10018581; doi:10.1007/s11481-023-10058-x)
Supplement: Supplementary file 1 — Supplementary file1 (PPTX 7687 KB) [file 11481_2023_10058_MOESM1_ESM.pptx]

## Slide 1
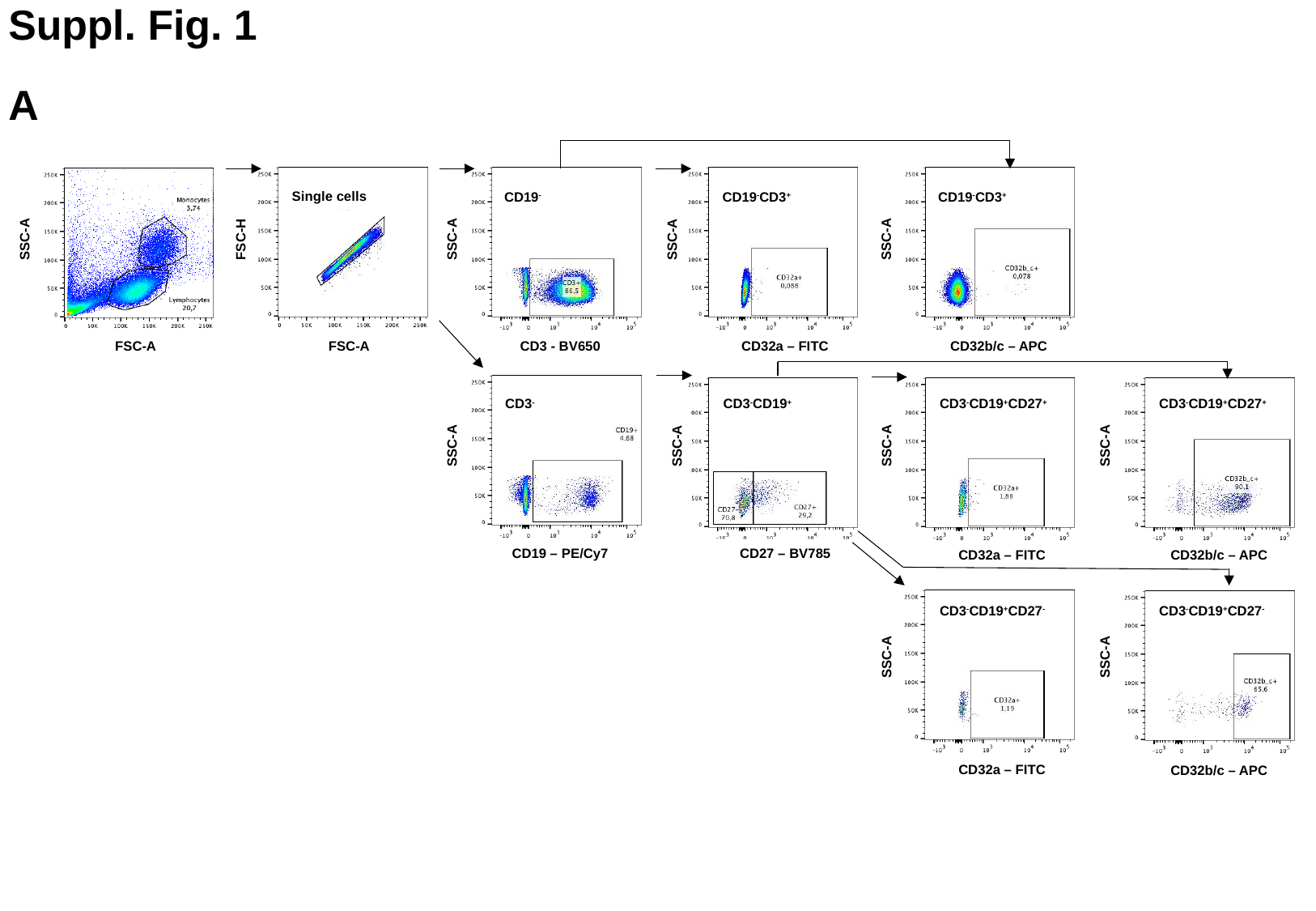

Suppl. Fig. 1
A
Single cells
CD19-
CD19-CD3+
CD19-CD3+
SSC-A
FSC-H
SSC-A
SSC-A
SSC-A
FSC-A
FSC-A
CD3 - BV650
CD32a – FITC
CD32b/c – APC
CD3-
CD3-CD19+
CD3-CD19+CD27+
CD3-CD19+CD27+
SSC-A
SSC-A
SSC-A
SSC-A
CD19 – PE/Cy7
CD27 – BV785
CD32a – FITC
CD32b/c – APC
CD3-CD19+CD27-
CD3-CD19+CD27-
SSC-A
SSC-A
CD32a – FITC
CD32b/c – APC

## Slide 2
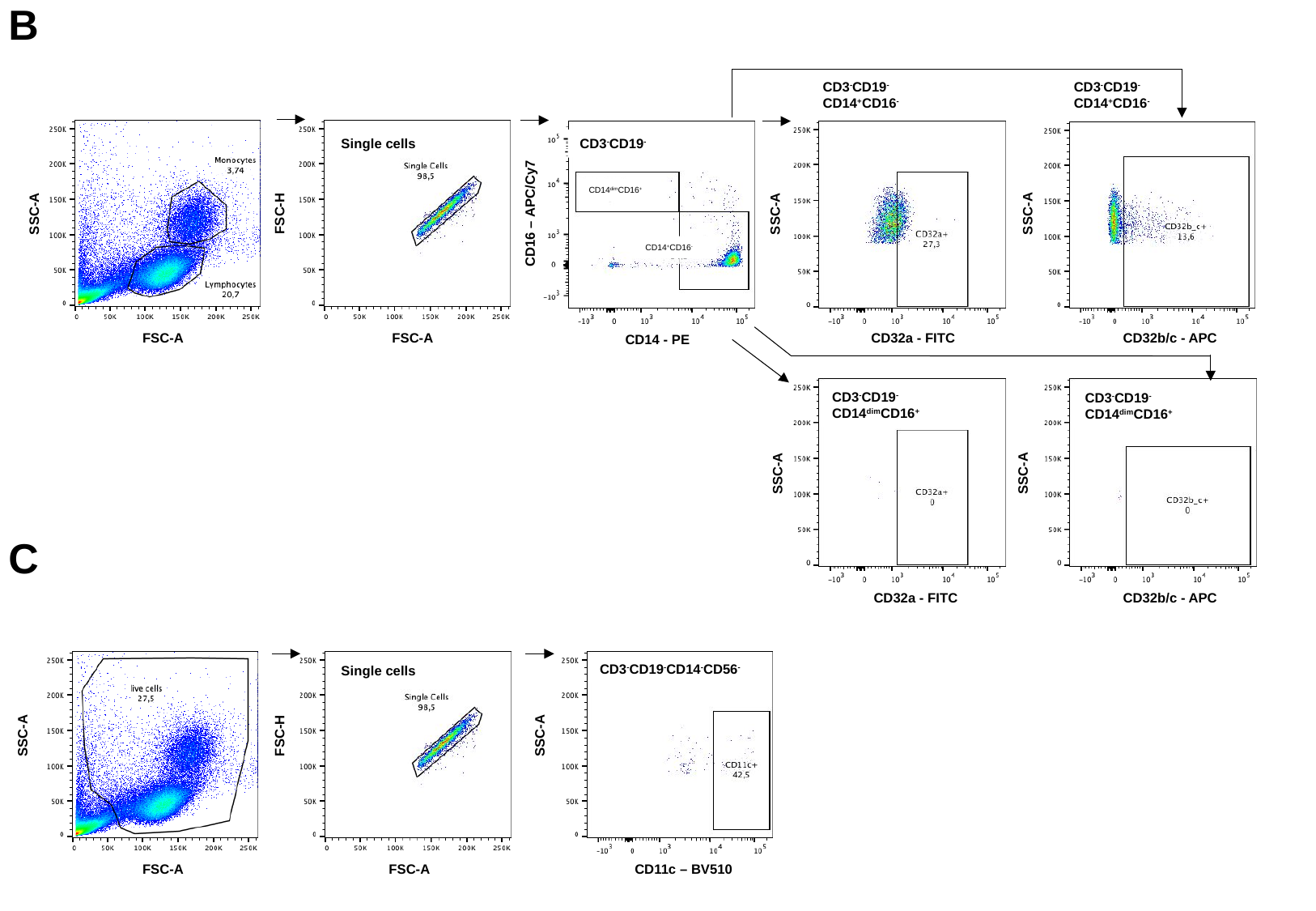

B
CD3-CD19-CD14+CD16-
CD3-CD19-CD14+CD16-
Single cells
CD3-CD19-
CD14dimCD16+
FSC-H
CD16 – APC/Cy7
SSC-A
SSC-A
SSC-A
CD14+CD16-
FSC-A
FSC-A
CD32a - FITC
CD32b/c - APC
CD14 - PE
CD3-CD19-CD14dimCD16+
CD3-CD19-CD14dimCD16+
SSC-A
SSC-A
C
CD32a - FITC
CD32b/c - APC
CD3-CD19-CD14-CD56-
Single cells
SSC-A
FSC-H
SSC-A
FSC-A
FSC-A
CD11c – BV510
